# Observations on the Origin, Symptoms, and Progress of the Plague, as It Appears at Constantinople

**Published:** 1806-02

**Authors:** 


### Observations on the Origin, Symptoms, and Progress of the Plague, as it appears at Constantinople.

( *From Pouqueville's Travels in the Morea, lately published at Paris.* )

WITH respect to the Plague, its very name implies the most terrible of disasters. In Asia, Africa, and even in the midst of the fortunate Isles of Greece, it manifests itself by general disease and death. Yet its nature and principles are enveloped in the deepest obscurity; and it may be considered as an emanation of celestial vengeance. In modern Greece, however, it is not Apollo who punishes an innocent people for the faults committed against the King of Kings, but a prejudice equally deplorable from the fear which it excites, renders the body susceptible of contagion.

The evil spirit, or cacodaimon, has been seen to glide along their roofs: no one dares to doubt the assertion; he is a decrepit object, covered with funeral shreds, and has been heard to call by their names, those whom he wished to cut off from the number of the living. Nocturnal music and murmuring voices have been heard in the air in the darkest nights, and phantoms have been seen moving in solitary places near the cemeteries. Strange dogs have howled in a dismal manner, and their voices have been terrifically re-echoed along the deserted streets.—Thus observed to me an inhabitant of Naupli, You must take care not to answer, if you hear yourself called in the night; you will sometimes be attracted by symphonies: do not listen to them, but cover yourself over in the bed; for it is the decrepit demon, that is, the Plague, which knocks at your door.

These ridiculous fears, from their frequent repetition, shake even the best-informed minds; and even grave historians have taken pleasure in representing such signs as fore-runners of the plague; and in consequence of these prejudices no simple description of the disease is yet known amongst the Greeks. I shall therefore endeavour to trace it, without embarrassing the reader with technical terms, and shall add the result of my own observations on the subject.

The nature of the plague is as little known as that of other diseases. To attribute it to effluvia or contagion is saying nothing, and throwing an obscurity on a point of the question which is not essential. I shall argue more to the purpose purpose by saying, that the plague arises from the insalubrity of certain situations and the impurity of the air. This was the opinion of Hippocrates; for, according to that Father of Medicine, the proximate cause of every disease is the air, which in proportion to its rarified or condensed state, contains morbific principles that penetrate with it into the human body. In fact, in the countries of Africa, for example in Egypt, where the plague is endemic, it always appears with the hot and moist winds from the south; and when the north winds commence, its ravages cease. From a constant appearance of this phenomenon, as given by every observer, it is not improbable that the plague is a destructive emanation from the Samm or wind of the desert, which Bruce describes as killing by a stroke of lightning.

The plague, however, was not known in Egypt in the early ages, notwithstanding the prevalence of the desert winds. Its ravages are not mentioned by Herodotus; nor was it known while that province was a Roman colony; but as soon as it fell to the weak Emperor of the East, who let every thing go to destruction, as soon, in short, as Egypt was invaded by the furious Amrou, the Lieutenant of the Caliph Omar, this fine country became the residence of the plague.

We ought not indeed to believe the assertions of Thucydides, Lucretius, and Pliny, that the plague proceeds from Ethiopia. Bruce, who travelled through Abyssinia does not mention its existence at Axum. The caravans which proceed every year from the interior of Africa, had spread it in Upper Egypt, before it was known at Cairo; but the contrary now takes place, for it comes from Lower Egypt, where it seems to be concealed in the environs of Damietta, and is propagated by contact. From the time of Procopius, it has appeared in a similar manner, as is evident from his description of a pestilence which spread over all the known country. "It began, says he, in Egypt, amongst the inhabitants of Pelusium, and gradually extended itself to Alexandria, in the other provinces, and in those parts of Palestine which are nearest to Egypt." Prof. Desgenettes makes the same remark when stating that the epidemy, by which he means the plague, appeared at Damietta in the month of September, and afterwards in the Marine Hospital at Alexandria.

The opinion of travellers, who pretend that the plague is conveyed to Constantinople and Egypt, by ships, is absurd. We have too many facts not to overthrow the assertion, were they they only in the numbers of our soldiery, who were destroyed by the plague in Egypt, at a time when all communication with Constantinople was cut off. But it may be said, that the fortunate territories of the East, the Grecian provinces subjected to the power of the Turks, are afflicted with this calamity from time to time; it, however, arises from the lakes of Albania and the Morea, together with the ruins of so many towns, which cause exhalations that favour its developement.

It is necessary to place amongst the fables and popular falsehoods, what is recounted of the signs that announce the plague. Thus epizooties, which are sometimes similar to the epidemy, and not essentially combined with it; the myriads of frogs and insects; the inundations; the hydrophobia, a disease well known in all the East; the spots of oil upon the walls, and the falling of meteors, all of which are asserted to be indications of the plague, are so many inventions proper for a novel, but which the impartial traveller ought only to mention in terms of ridicule.*

The most certain sign of the plague is the hot and moist constitution mentioned by Hippocrates. Constantinople and the whole of Greece being under the influence of such an atmosphere, are always liable to the plague; and it may be said with Lucretius, that the germs of this disease cross the sea in the air, and descend on the people of Pandion. This is the period to shut oneself up, as such means are considered as an infallible preservative. The pestilence immediately attacks the people who walk abroad, and are ignorant of their danger; yet vegetation is never more beautiful than at such a period; the corn is attacked with a kind of smut, but the meadows are every where enamelled with flowers, which even grow amongst the cypress of the tombs.

The commencement of the plague may now be easily ascertained from the appearance of the first victims who have been struck with it. Its symptoms are as follow, cardialgia, bitterness of taste, head-ache, lassitude, horripilation which frequently occurs towards evening, are the common symptoms of all malignant or adynamic fevers, but shiverings, anorexia, sinking of the pulse, sleeplessness or rather sleep accompanied with frightful dreams, and a

 melancholic

* With respect to the hydrophobia, many creditable persons have assured me that mad dogs have been seen at Constantinople; but notwithstanding their veracity, I positively assert the contrary.

melancholic habit, seem to be more nearly allied to the pestilential or adeno-nervous fever, which is only the putrid fever in the highest degree.

An attack of the plague is never so sudden as to cause men to fall down in the streets, as if they were struck by lightening. The only people who perish in this manner are such as are without a home; and this class is very common in the East, where almost the whole year, and particularly at the time of the plague, the unfortunate men lie under or on the top of the warm ashes from the public baths. Their miserable mode of life renders them the earliest victims to the epidemy; and in all cases, those who have died rather suddenly, have previously exhibited some of the symptoms of a pestilential fever.

The first patients who are attacked by the plague, generally leave an uncertainty, as to the nature of the malignant fever which prevails; it is, however, known to have three stages, during which it assumes different characters; though sometimes, when it exerts its greatest ravages, it exhibits them all at once.

With some persons the approach of death is indicated by vomitings, cephalalgia, feeble pulse, and large black spots; they in general die soon after, when the limbs preserve their flexibility; and in a few hours the corpse exhales an insupportable smell. But the most unfortunate beings are the women in child-bed, who quickly receive the infection, and never escape its consequences. Those who are previously weakened by violent fevers, or acute diseases, likewise fall a prey to the first attacks of the plague.

Some patients are afflicted with delirium, raving madness, or a burning fever; their tongue is red, dry, and cracked; their eyes are sparkling, and sometimes filled with tears; and their looks are altogether singular. The bubo does not appear till the moment of death, when it often rises under one of the arm-pits or on the breast. Others are afflicted with a pestilential angina; and the fauces and larynx are inflamed by numerous ulcers which impede respiration, and, at first, make the patient appear to be attacked by the croup. A cadaverous stench issues from the mouth; the tongue is covered with a blackish sanies; and the tumefied lips give the unfortunate beings a horrid appearance. They complain of a parching thirst, feel as if a fire was burning within them, and generally die on the fifth day. The plague is mild when it follows the progress of putrid or adynamic fevers; the bubo, which, however, however, is not one of its essential characteristics, appears between the fourth and fifth day, and always in the groin, or on the thigh; it soon suppurates; the tongue and teeth which, till then, had been black, become white; the patient recovers his senses, and his hopes revive, particularly on finding that he is not abandoned by his relatives. If the bubo be long in arriving at suppuration, his convalescence is tedious and violent; and the patient for years afterwards feels pain on the return of the epidemic season.

Being terrible at its commencement, more from the consternation which it spreads than from the evils it occasions, the plague seems to be propagated by the alarm which every one feels; and thus, by adducing debility, renders himself susceptible of its attacks. On the least suspicion of the plague, the most courageous men become depressed, alarmed, and seriously ill; while many, from this predisposition alone, have been attacked by it; hence the fear of death precipitates them to the tomb.

On reaching its second stage, the plague covers the cities with funerals. The silence of the night is only interrupted by groans, and the plaintive cries of the dying, combined with the lamentation of whole families who are stricken by the contagion; very few escape without lingering for the remainder of their lives in a deplorable state. The streets are abandoned, the people avoid each other, and they dare not ask any questions, lest they should hear of the loss of a parent or friend. At this period of affliction the Turk who resides in Constantinople begins to believe in the existence of the plague, as nearly a thousand corpses are conveyed in a single day through the gate of Adrianople. These mortuary processions give the rallying signs to the Mussulmen, who meet in the plains of Okmeidan, to invoke the Divinity to stay his wrath; they do not complain of their losses, for *God hath willed them*; they merely intreat a cessation of the calamity, and pray to be made whole.

In this general mourning the Mussulman, blinded by fate, sees in the plague which devours him, nothing but one of the irrevocable decrees of heaven; although he does not blame the Greek for being alarmed, or the Frank for being shut up, he believes himself to have sinned by wanting confidence; and that, if Providence has so decreed it, his prayers will be heard; for he is convinced that his days are numbered, and his fate decreed from all eternity; and in this he is not stupid or apathetic, but religious. His children and wives perish before him, his heart bleeds,

 he he sheds the most afflicting tears, and bows his head to Providence, who has overwhelmed him; he remains in his house, gives his orders coldly, and performs the duties of his religion in his usual dress. But death still continues his ravages, and the Turk at length remains at home like an ancient tree in the midst of a forest, devastated by the winds. He raises his hands towards Heaven, in which he sees his country, and observes, that this world is a place of passage. In short, he dies in his turn, but without having undergone pusillanimous agony and fear, a hundred times worse than death itself. This second period is the crisis of the pestilential disease; children, women, and weak men mostly fall victims to it; but fortunately it is of short duration. On arriving at its third stage there is a remission of its principal characters of malignity; it no longer follows the ataxic progress, as in the second period; nor does it screen itself under the mask of other diseases, but appears openly, and assumes a decisive form; the patient no longer has ulcers, spot, or sore throat; but the bubo is the prevalent symptom. In this stage a greater number of patients escape; while infants and youth are almost the only victims; in short, it disappears, as the temperature changes, or when the cold is first felt in Europe.

The plague commits such extensive ravages only at distant intervals. It is believed that it returns with increased force at Constantinople once in nine years; but it never appears there, when the communication with Egypt is interrupted by war; nor is it so absolutely contagious as the Franks, who inhabit the Levant, would make us believe; for if so, how few of our eastern army would have returned to their country?

On reflecting on the pestilential fever, I dare not hope for the discovery of a specific, as it is often difficult to distinguish this Protean malady; so many fruitless attempts have been made, and such a number of remedies proposed for the plague that it is even ridiculous to speak of them. Seduced by the idea that it was occasioned by a particular and homogenous virus, M. Valli lately made an attempt, as bold as it was interesting. He thought he had discovered, about two years ago, that persons who had been vaccinated, were not attacked by the pestilential disease which then prevailed at Constantinople; he therefore concluded, that the vaccine virus would neutralize, what he called, the pestilential virus, as Dr. Swediaur had proved that mercury, combined with the pus from a syphilitic tumour, destroyed its contagion. He therefore took some some pus from the bubo of a person attacked by the plague, and mixed it with a certain quantity of vaccine virus, with which he had the courage to inoculate himself. No inconvenience resulted from the experiment; but what conclusions ought to be drawn from a single instance? Certainly we ought not to give credit to the insignificant reports which were afterwards circulated. Let us rather praise the wisdom of modern governments, who have placed barriers against the plague by the establishment of lazarettoes, and a performance of quarantine; while culture and civilization have destroyed its first principles We may add that an European, who travels through, or resides in, the Turkish empire, should adopt means for his preservation, the first of which is courage; while the life of pleasure, mentioned by Boccacio, and a sort of demi-epicuranism are excellent preventatives. Wholesome food and moderate exercise should be adopted as much as possible: and these with a degree of confidence, not extended to fool-hardiness, will save him from danger. As to the physician, his duty is evident from the engagement which he has made to assist the unfortunate; he need not to go and sit on their pillows; but he should appear, like the minister of peace in days of mourning; like the desired angel in the midst of horror stricken families, to whom he should give hopes, that would enable them to muster strength to resist the disease; and if his hour be fixed, as he must die at last, he will meet with an end appropriate to his zeal, and expire in the midst of good works.

